# Evaluation of clinical and laboratory manifestations related to the gastrointestinal system and prognosis in hospitalized patients with COVID-19: a prospective cohort study

**DOI:** 10.1186/s12879-026-12814-9

**Published:** 2026-02-13

**Authors:** Thiago Henrique Fernandes de Carvalho, Fabyan Beltrão Esberard, Maryana Cavalcanti Holanda, Matheus Venâncio de Paiva, Daniele Carvalhal de Almeida Beltrão, Giulia Carvalhal, José Felipe Lacerda Fernandes, Carlos Alexandre Antunes de Brito

**Affiliations:** 1 Postgraduate Program in Tropical Medicine, Federal University of Pernambuco, Recife, Pernambuco Brazil; 2 Department of Internal Medicine, Lauro Wanderley University Hospital, João Pessoa, Paraíba Brazil; 3 Department of Internal Medicine, Alberto Urquiza Wanderley Hospital, João Pessoa, Paraíba Brazil; 4 Postgraduate Program in Cognitive Neuroscience and Behavior, Center for Health Sciences, Federal University of Paraíba, João Pessoa, Paraíba Brazil; 5 Center for Biological and Health Sciences, Federal University of Campina Grande, João Pessoa, Paraíba Brazil; 6 School of Medicine, João Pessoa University Center, João Pessoa, Paraíba Brazil; 7 Postgraduated Program in Tropical Medicine, Federal University of Pernambuco, Recife, Pernambuco Brazil; 8 Postgraduate Program in Tropical Medicine, Federal University of Pernambuco, 580 Norberto de Castro Nogueira Avenue, João Pessoa, Paraíba 58037-603 Brazil

**Keywords:** COVID-19, Gastrointestinal diseases, Liver function tests, Prognosis, Brazil, Cohort studies

## Abstract

**Background:**

The prognostic implications of gastrointestinal symptoms and abnormal liver enzymes in COVID-19 patients have shown significant variability. This study aimed to describe the incidence of these manifestations and their correlation with disease severity in a Brazilian population during the early phase of the pandemic, a context with limited published data.

**Methods:**

This was a prospective cohort study of 253 consecutive patients with SARS-CoV-2 conducted in a tertiary hospital in João Pessoa, Brazil. Patients were evaluated for the presence of gastrointestinal symptoms, elevated liver enzymes, and clinical outcomes (ICU admission, mortality). Statistical analysis included Mann-Whitney, chi-square, or Fisher tests, and logistic regression. Inclusion criteria were a positive rRT-qPCR for SARS-CoV-2 or clinical-radiological findings (CO-RADS 5) with positive serology. Patients were classified as severe or critical based on respiratory rate, oxygen saturation, and organ failure requiring mechanical ventilation or ICU care.

**Results:**

Forty-nine (19.37%) patients presented with gastrointestinal symptoms. No significant differences were found in ICU admission (20.4% vs. 24.2%, *p* = 0.707) or mortality (16.2% vs. 18.3%, *p* = 0.674) between groups with and without these symptoms. Elevation of liver enzymes during hospitalization was associated with a longer hospital stay (median 7 days vs. 5 days, *p* = 0.0016) but not with ICU admission or mortality.

**Conclusions:**

In this cohort, gastrointestinal symptoms and elevated liver enzymes at admission were not predictors of mortality. However, in-hospital liver enzyme elevation was associated with a longer hospital stay, highlighting its importance as a marker for resource management rather than mortality risk.

**Clinical trial number:**

Not applicable.

## Introduction

Coronavirus disease 2019 (COVID-19), caused by the novel coronavirus (SARS-CoV-2), has reached nearly 770 million cases worldwide. Among these, 38,743,918 cases have been reported in Brazil, with more than 711,380 deaths as of April 2024 [1]. The northeastern region of Brazil was the second most affected region in the country during the initial period of the pandemic [2, 3]. To combat this disease, research has been conducted in various regions around the world. Initial studies aimed at describing the clinical characteristics of patients infected with the new coronavirus 2019 (SARS-CoV-2) reported a low incidence of symptoms and laboratory abnormalities related to the digestive system. A study by Huang et al. (2020) reported the presence of diarrhea in only three percent of affected individuals. However, in a retrospective study conducted in New York, reported a considerably higher incidence of diarrhea (23.7%), as well as nausea and vomiting in 19.1% of patients diagnosed with COVID-19 [4]. However, that study did not correlate the presence of these symptoms with disease severity. Such differences, possibly due to genetic, environmental, and socioeconomic variables, highlight the importance of more localized studies considering the abovementioned characteristics as well as the context in which the data were collected, including the study design [5–7].

In this context, Jin et al. (2020), when analyzing changes in AST (aspartate aminotransferase) and ALT (alanine aminotransferase) levels, reported a correlation between the presence of GI symptoms and elevated serum AST levels. However, many questions about this association remain, as few studies have established a significant relationship between the presence of GI symptoms, hepatic changes, and progression to more severe forms of the disease [8]. However, some evidence suggests that elevated liver enzymes in patients with COVID-19 are not necessarily associated with disease severity but rather with the direct action of the virus and treatment-related factors [9].

Therefore, studies assessing epidemiological and clinical characteristics in different scenarios are crucial not only for a better understanding of the virus and the disease associated with it but also for providing consistent information to help adjust parameters that may integrate prognostic models. However, clinical studies evaluating these aspects in Brazil, especially in the northeastern region, are still rare and are the focus of this research.

The primary objective of this study was to evaluate the incidence of gastrointestinal (GI) symptoms and liver enzyme (LE) alterations in a cohort of 253 hospitalized COVID-19 patients and to analyze their association with disease severity and clinical outcomes (ICU admission and mortality)

## Materials and methods

### Subjects and data collection

**Fig. 1:**
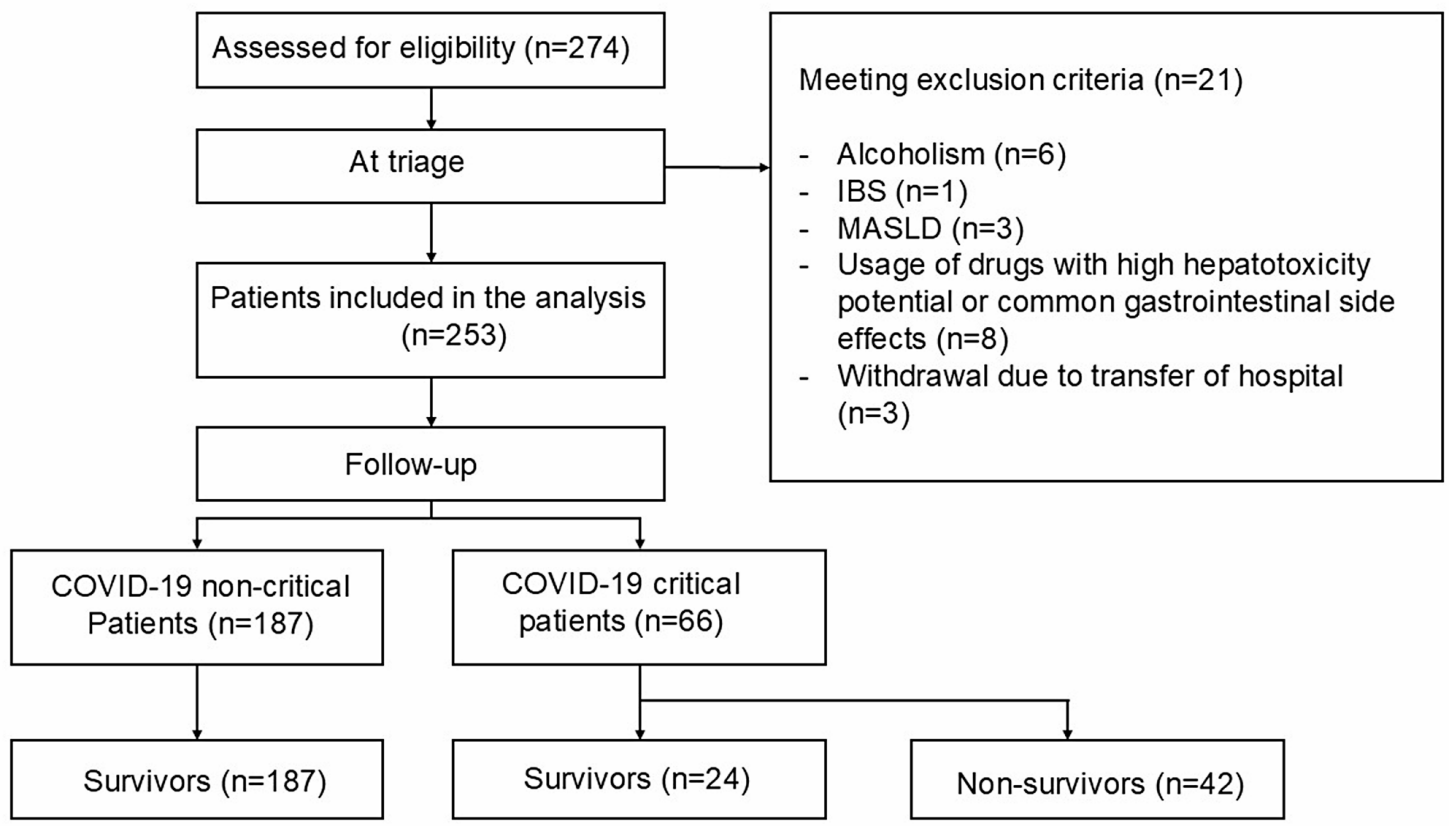
Flowchart of the study participant selection process. IBS, irritable bowel Syndrome; MASLD, metabolic dysfunction-associated steatotic liver disease

A longitudinal observational and prospective cohort study was conducted between June and August 2020, including 253 consecutive patients with confirmed COVID-19 admitted to the Dom José Maria Pires Metropolitan Hospital, a tertiary referral hospital in João Pessoa, Paraíba, Brazil (Fig. 1). Written informed consent was obtained from the participants or their legal representatives. The study was approved by the Human Research Ethics Committee of the João Pessoa University Center (CAAE: 50,361,021.80000.5176). Figure 1 shows the flowchart of the study.

### Inclusion and exclusion criteria

The inclusion criteria for patients were as follows: a positive test for SARS-CoV-2 via quantitative real-time reverse transcription polymerase chain reaction (rRT‒qPCR) with respiratory tract samples; or, in cases with a negative rRT-qPCR result, patients who met clinical, radiological (i.e., ground‒glass opacities with or without consolidation located near the visceral pleural surface and bilateral multifocal distribution—CO-RADS 5), and serological (i.e., IgG positive for SARS-CoV-2) criteria. The rRT‒PCR kit used was Biomol OneStep/COVID-19 IBMP Paraná Brazil. Exclusion criteria: Patients with a history of gastrointestinal disease, chronic alcoholism, a diagnosis of acute or chronic liver disease, or those taking medications with known hepatotoxicity or common gastrointestinal side effects were excluded.

### Procedures

Detailed clinical information for each patient was obtained by physicians via a standard questionnaire, previously developed and utilized by our research group [10], which assessed the presence of gastrointestinal symptoms, among other data. Two severity scoring systems were used at admission: (i) the Sepsis-Related Organ Failure Assessment (SOFA) and (ii) the National Early Warning Score 2 (NEWS2). Patients underwent chest computed tomography at hospital admission to investigate suspected SARS-CoV-2 pneumonia. All patients were classified into two clinical categories: severe and critical. Patients were classified as having severe (noncritical) disease if they met any of the following criteria: respiratory rate greater than 30 cycles/min, oxygen saturation below 93% at rest, partial pressure of oxygen (PaO2)/oxygen concentration (FiO2) below 300 mmHg (1 mmHg = 0.133 kPa), and estimated lung lesion extent (ground-glass opacity) greater than 50%. Patients were classified as having critical disease if they met any of the following criteria: manifestation of respiratory failure requiring mechanical ventilation, presence of shock, and other organ failure requiring intensive care unit (ICU) monitoring and treatment. For patients meeting the inclusion criteria, blood samples were collected prior to interventions or therapies that could interfere with or alter serum liver enzyme levels, within the first 48 hours of admission and subsequently as needed, with the maximum value during hospitalization being evaluated.

### Serum biochemistry

Complete blood cell counts and measurements of lymphocyte and neutrophil subpopulations were performed via a MEK-7300 hematology analyzer (Nihon Kohden Tokyo Japan). Alanine transaminase (ALT), aspartate transaminase, creatinine, high-sensitivity C-reactive protein (CRP), D-dimer, lactate dehydrogenase (LDH), and ferritin were measured via chemiluminescence immunoassay (MAGLUMI-2000-PLUS; Shenzhen New Industries Biomedical Engineering Co. Shenzhen China) according to the manufacturer’s protocol.

### Statistical analyses

A statistical power analysis was performed to estimate the sample size. The effect size in this study was conservatively selected as f2 = 0.10. With alpha = 0.05 and power = 0.95, the projected sample size needed with this effect size using GPower 3.1.9.7 is approximately *N* = 158 for a linear regression analysis with two predictors. Thus, our sample size of 253 was more than adequate for the primary outcome of this study and should allow for expected attrition. Data are expressed as the median and interquartile range. Mann‒Whitney, chi‒square, or Fisher tests were used for nonparametric variables. To assess the relative risk of mortality, we used univariate and multivariate logistic regression. We evaluated each variable as a potential biomarker via receiver operating characteristic (ROC) curves. A significance level of *p* < 0.05 was accepted as statistically significant. GraphPad Prism v0.7.00 (2016) statistical software was used to perform the statistical tests.

Laboratory variables were compared between patients with and without GI symptoms, and between those with and without elevated liver enzymes. To identify independent predictors of hospital mortality, a multivariate logistic regression analysis was performed, adjusting for age and clinical severity.

## Results

Two hundred seventy-four adult patients who were consecutively admitted with COVID-19 were considered for potential inclusion in the study, and after the inclusion and exclusion criteria were evaluated, 253 patients were enrolled (Fig. 1). The median age was 63 years (50–74) (*p* = 0.235), and 149 patients (58.9%) were male. The median hospital stay was 6 days. In terms of severity, 187 patients were classified as noncritical (73.91%), and 66 patients were classified as critical (26.09%). Fifty-nine patients (23.4%) were admitted to the ICU, 42 (16.6%) of whom died. Table 1 summarizes the sociodemographic and clinical characteristics of the patients analyzed and their correlation with mortality.

**Table 1:** Sociodemographic and clinical characteristics of 253 patients hospitalized with COVID-19 in João Pessoa, Brazil (June–August 2020), categorized by clinical severity and mortality

Variables	Total (*n* = 253)	Non critical (*n* = 187)	Critical (*n* = 66)	P Value	Survivours (*n* = 211)	Non survivours (*n* = 42)	P Value
Age (years), median (IQR)	63 (50–74)	62 (49–74)	64 (50–75.75)	0.1842	62 (49–74)	63.5 (50.5–76.75)	0.235
Age > 60 years, n (%)	139 (54.9%)	98 (52.4%)	41 (62.1%)	0.1965	112 (53.1%)	24 (57.1%)	0.7352
Male sex, n (%)	149 (58.9%)	113 (60.4%)	36 (54.5%)	0.4673	126 (59.7%)	23 (54.8%)	0.6078
Severity (Critical), n (%)	-	-	-	-	24 (11.4%)	42 (100%)	< 0.0001
Comorbidities							
Hypertension, n (%)	169 (66.8%)	120 (64.2%)	49 (74.2%)	0.1711	140 (66.4%)	29 (69%)	0.858
Diabetes mellitus, n (%)	110 (43.7%)	78 (41.7%)	32 (49.2%)	0.3121	90 (42.7%)	20 (48.8%)	0.6105
Cardiopathy, n (%)	35 (13.8%)	27 (14.4%)	8 (12.1%)	0.8359	33 (15.6%)	2 (4.8%)	0.0841
Neoplasia, n (%)	2 (0.8%)	1 (0.5%)	1 (1.5%)	0.4529	1 (0.5%)	1 (2.4%)	0.305
Chronic lung disease, n (%)	11 (4.3%)	10 (5.3%)	1 (1.5%)	0.2974	10 (4.7%)	1 (2.4%)	0.6971
Complications							
Use of vasoactive drugs, n (%)	31 (12.4%)	1 (0.5%)	30 (45.5%)	< 0.0001	6 (2.9%)	25 (59.5%)	< 0.0001
ICU admission, n (%)	59 (23.3%)	0 (0%)	59 (89.4%)	< 0.0001	24 (11.4%)	35 (83.3%)	< 0.0001
Hospital mortality, n (%)	42 (16.6%)	0 (0%)	42 (63.6%)	< 0.0001	-	-	-
Duration of hospitalization (days), median (IQR)	6 (4–10)	5 (4–7)	11 (7.25–17)	< 0.0001	6 (4–8)	12.5 (8–17)	< 0.0001
Severity scoring systems							
NEWS2 Score, median (IQR)	6 (5–7)	6 (4–7)	5 (5–7)	0.4195	6 (4.5–7)	6 (5–7)	0.3144

IQR, interquartile range; ICU, intensive care unit; NEWS2, National Early Warning Score 2. *p* values were calculated using the Mann-Whitney test for continuous variables and Fisher’s exact test for categorical variables

The Mann‒Whitney test was performed for continuous variables (age, length of hospital stay, and scoring system), whereas Fisher’s exact test was performed for all other variables.

### Presence of gastrointestinal symptoms

Upon admission, 49 out of 253 patients evaluated presented gastrointestinal (GI) symptoms (19.37%). The median age of patients with GI symptoms was 61 years (*p* = 0.878). The group with GI symptoms had a significantly lower proportion of male patients than the group without GI symptoms (38.8% vs. 63.7%, *p* = 0.002). The severity score (NEWS2) within the first 48 hours was similar between the groups with and without gastrointestinal symptoms: the median score was 6 (IQR: 5–7) and 6 (IQR: 4.5–7.5), respectively (*p* = 0.246).

Patients with GI symptoms had a slightly longer median hospital stay of 7 days (IQR 4–14.5), whereas patients without GI symptoms had a median hospital stay of 6 days (IQR 4–10) (*p* = 0.320). ICU admission occurred in 10 (20.4%) of the 49 patients with gastrointestinal symptoms compared with 49 (24.2%) of the 204 patients without digestive symptoms (*p* = 0.707) (Table 2).

**Table 2:** Comparison of median laboratory values upon admission between patients with (*n* = 49) and without (*n* = 204) gastrointestinal symptoms

Variables (normal range)	Total (*n* = 253)	GI Symptoms Present (*n* = 49)	GI Symptoms Absent (*n* = 204)	P value
Hemoglobin (13–18 g/dL)	13.4 (12.3-14.4)	13.0 (12.1–13.7)	13.5 (12.4–14.5)	0.036
INR (0.8–1.0)	1.0 (1.0–1.06)	1.0 (1.0–1.06)	1.0 (1.0–1.07)	0.37
Albumin (3.5–5.5 g/dL)	3.3 (2.9–3.6)	3.3 (2.9–3.7)	3.3 (2.9–3.6)	0.682
TB (0.2–1.1 mg/dL)	0.37 (0.28–0.49)	0.30 (0.25-0.55)	0.37 (0.29–0.48)	0.335
AST (8-42 U/L)	54 (38-80)	52 (36-80)	57 (42-92)	0.214
ALT (8-42 U/L)	61 (39- 98)	58 (38-90)	62 (39-101)	0.768
CRP ( < 5.0 mg/dL)	95 (39-152)	105 (41-171)	93 (35-151)	0.497
LDH (207-414 U/L)	740 (551-1009)	754 (573-1051)	740 (550-1007)	0.769
D-dimer ( < 500 ng/mL)	785 (500-1628)	834 (486- 2374)	775 (517-1506)	0.866
H1bc (4% − 5.6%)	7.5 (6.7–9.2)	7.7 (6.9-9.7)	7.4 (6.6–8.9)	0.272

Values are presented as median (interquartile range). INR, international normalized ratio; TB, total bilirubin; AST, aspartate transaminase; ALT, alanine transaminase; CRP, C-reactive protein; LDH, lactate dehydrogenase; HbA1c, glycated hemoglobin. *p* values were calculated using the Mann-Whitney test

**Fig. 2:**
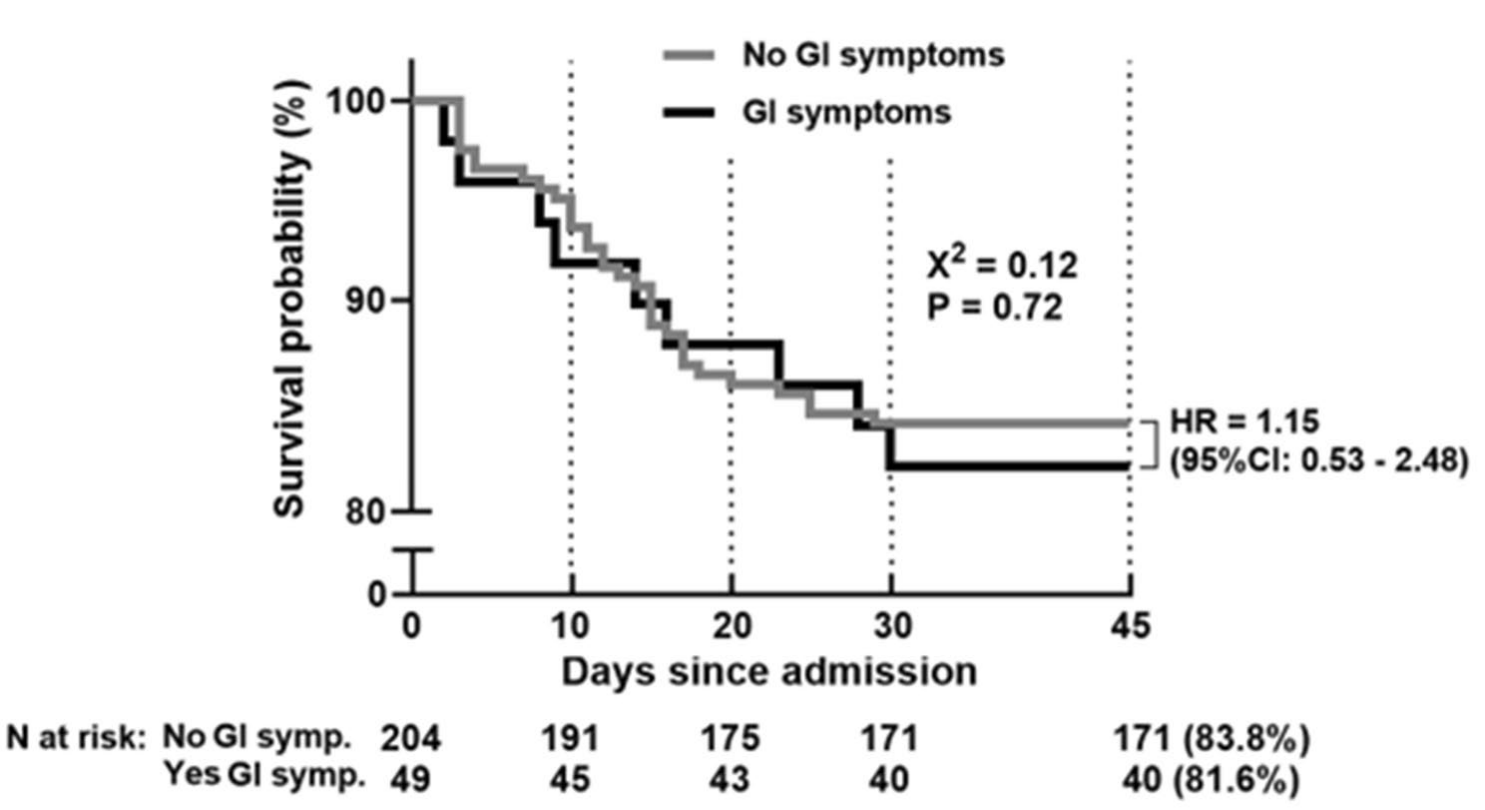
Kaplan‒Meier survival curve comparing patients with and without gastrointestinal symptoms over 45 days. GI, gastrointestinal; HR, hazard Ratio; CI, confidence interval

There were no significant differences in mortality between the GI symptoms group, with 9 deaths (16.2%), and the group without gastrointestinal symptoms, in which 33 patients died (18.3%) (*p* = 0.674). There was no significant difference in 45-day survival between patients with gastrointestinal symptoms (81.6%, *n* = 49) and those without gastrointestinal symptoms (83.8%, *n* = 204) (HR = 1.15; 95% CI: 0.53–2.48; *p* = 0.72) (Fig. 2).

### Laboratory data

The laboratory data revealed that patients with gastrointestinal symptoms presented slightly lower hemoglobin levels than did those without gastrointestinal symptoms, with values of 13.0 g/dL (12.1–13.7) versus 13.5 g/dL (12.4–14.5), which was statistically significant (*p* = 0.036). Transaminase levels were similar between the two groups. In the group with gastrointestinal manifestations, the values were 58 (38-90) U/L for ALT and 52 (36-80) U/L for AST, whereas in the group without gastrointestinal manifestations, the values were 62 (39-101) U/L for ALT and 57 (42-92) U/L for AST. The differences were not statistically significant (*p* = 0.768) for ALT and (*p* = 0.214) for AST.

The total bilirubin level (TB: NR 0.2–1.1 mg/dL) was 0.30 (0.25–0.55) mg/dL in the group with gastrointestinal symptoms versus 0.37 (0.29–0.48) mg/dL in the second group (*p* = 0.335). The average albumin concentration (NR 3.5–5.5 g/dL) was identical in both groups: 3.3 (2.9–3.7) g/dL versus 3.3 (2.9–3.6) g/dL (*p* = 0.682). Similarly, the INR (NR 0.8–1.0) was 1.0 (1.0–1.06) in the gastrointestinal symptoms group and 1.0 (1.0–1.07) in the non-gastrointestinal symptoms group (*p* = 0.37).

The D-dimer level (NR < 500 ng/mL) was 834 (486–2374) ng/mL in the group with gastrointestinal manifestations and 775 (517–1506) ng/mL in the group without these manifestations (*p* = 0.866). LDH (NR 207-414 U/L) presented averages of 754 (573-1051) U/L in the group with gastrointestinal manifestations and 740 (550-1007) U/L in the group without gastrointestinal manifestations (*p* = 0.769). CRP (NR < 5.0 mg/dL) was 105 (41-171) mg/dL in the group with gastrointestinal manifestations and 93 (35-151) mg/dL in the group without these manifestations (*p* = 0.497). H1bc levels (NR 4% − 5.6%) were 7.5 (6.7–9.2) for all patients (*n* = 253), 7.7 (6.9-9.7) in the gastrointestinal symptoms group (*n* = 49) and 7.4 (6.6–8.9) in the gastrointestinal symptoms group (*n* = 204) (*p* = 0.272).

### Liver enzymes (LEs)

Among the patients included in the study, 35.6% presented with altered liver enzymes (LE) (*n* = 90), whereas 64.4% presented with levels within the normal range (*n* = 163). During follow-up, elevations in LE levels were observed in 45.8% of the patients (*n* = 116), whereas 54.2% continued with LE values within the reference parameters (*n* = 137).

Patients with altered LE had a significantly lower median age (55 years) than patients without altered LE, who had a median age of 65 years (*p* = 0.0053). The proportion of male patients with elevated LE was 67.8% (*n* = 61), which was significantly greater than that of patients with normal LE, which was 54% (*n* = 88) (*p* = 0.033).

The analysis of admission CRP ( < 5.0 mg/dL) between the groups with elevated LE and the group with normal LE revealed median values of 104 (33–170) in the group with altered LE and 93.8 (40–150) in the group with normal LE, without a statistically significant difference (*p* = 0.683). CRP showed similar behavior concerning the peak elevation of enzymes during hospitalization, with median values of 110.5 (35–176) in the group with LE above the reference values and 84 (40–149) in the group with normal LE, but the difference was not statistically significant (*p* = 0.334).

The evaluation of D-dimer ( < 500 ng/mL) revealed median values of 765 (495–1987) in the group with increased LE and 797 (507–1521) in the group with normal LE (*p* = 0.750). During hospitalization, D-dimer measurements revealed median values of 733 (500–1738) in the group with elevated LE and 829 (504–1566) in the group with normal LE, which was also not significantly different (*p* = 0.919). The assessment of albumin levels in the admission data regarding liver enzymes revealed a median of 3.3 (3.0–3.6) in the group with elevated LE and 3.3 (2.9–3.6) in the group with normal LE (*p* = 0.557). During the in-hospital follow-up, 3.3 (3.0–3.7) patients in the group that presented LE alterations and 3.3 (2.9–3.6) patients in the group with normal LE were measured, which was also not statistically significant (*p* = 0.451).

However, the comparative analysis of lactate dehydrogenase (LDH) levels revealed a statistically significant difference between patients with abnormal liver enzymes (LE) and those with normal LE both at admission and throughout hospitalization. At admission, patients with abnormal LE had average LDH levels of 834 U/L, which was significantly greater than that of patients with normal LE, who had average levels of 677 U/L (*p* < 0.0001). This trend persisted throughout the hospitalization period, indicating a possible correlation between serum transaminase levels and LDH values.

Additionally, there was a significant difference in hemoglobin A1c (HbA1c) levels between the groups with abnormal and normal LE. At admission, the group with abnormal LE presented average HbA1c levels of 7.1%, whereas the average HbA1c level was 7.9% in the group with normal LE (*p* = 0.007). During hospitalization, the levels continued to be significantly lower in the group with abnormal LE (7.1%) than in the group with normal LE (8.0%, *p* = 0.014). These findings suggest that, in the studied group, metabolic alterations did not appear to contribute to the observed variations in liver enzymes.

The neutrophil/lymphocyte ratio (N/L), although not statistically significant at admission (*p* = 0.153), was significantly different during hospitalization, with patients with abnormal LE presenting an average of 10.4 compared with 8.7 in the group with normal LE (*p* = 0.05)

The length of hospital stay was similar in both groups: 6 (4–10) versus 6 (5–10) (*p* = 0.830) at admission. However, the elevation of LE during hospitalization was associated with a longer hospital stay, with a median of 7 days (IQR: 5–12), than 5 days (IQR: 4–8.5) in patients who did not present this finding (*p* = 0.0016) (Table 3).

**Table 3:** Clinical and laboratory characteristics of 253 COVID-19 patients according to liver enzyme (LE) alterations at admission and peak values during hospitalization

Variables	LE admission					LE maximum value				
	Altered LE		Normal LE		p value	Altered LE		LE normal		p value
	**(n = 90)**		**(n = 163)**			**(n = 116)**		**(n = 137)**		
**Age (i), median (IQR)**	**55 (47–71)**		**65 (52–75)**		**0.0053**	**55 (47–70)**		**67 (53–76)**		**0.0003**
**Male gender, n (%)**	**61 (67.8)**		**88 (54)**		**0.033**	75 (64.6)		74 (54)		0.096
**Hospital stay (d), median (IQR)**	6 (4–10)		6 (5–10)		0.830	**7 (5–12)**		**5 (4–8.5)**		**0.0016**
**ICU admission, n (%)**	23 (25.5)		36 (22.1)		0.538	32 (27.6)		27 (19.7)		0.179
**Mortality, n (%)**	14 (15.5)		28 (17.1)		0.860	22 (18.9)		20 (14.6)		0.398
**Hemodialysis, n (%)**	29 (32.2)		62 (38)		0.412	39 (33.6)		52 (37.9)		0.512
**Use of vasoactive drugs, n (%)**	12 (13.3)		19 (11.6)		0.693	19 (16.3)		12 (8.7)		0.083
**NEWS2 Score, median (IQR)**	6 (5–7)		5 (4–7)		0.086	6 (5–7)		5 (4–7)		0.241
**CT COVID Severity Score, median (IQR)**	20 (15–20)		20 (15–20)		0.672	20 (15–20)		20 (12.5–20)		0.277
**IL-6 ( < 3.4 pg/mL)**	50 (24–105)		53 (20–95)		0.681	47 (23–102)		54 (20–95)		0.974
**D-dimer ( < 500 ng/mL)**	765 (495–1987)		797 (507–1521)		0.750	733 (500–1738)		829 (504–1566)		0.919
**LDH (207-414 U/L)**	**834 (685–1149)**		**677 (504–917)**		** < 0.0001**	**818 (638–1145)**		**677 (492–905)**		** < 0.0001**
**CRP ( < 5.0 mg/dL)**	104 (33–170)		93.8 (40–150)		0.683	110.5 (35–176)		84 (40–149)		0.334
**Neutrophils (1.9–6.7 10**^**3**^** cells/µL)**	7.7 (5.4–9.2)		7.3 (5.1–9.9)		0.911	7.8 (5.3–9.6)		7.1 (5.2–9.4)		0.672
**HbA1c (4% − 5.6%)**	**7.1 (6.5–8.2)**		**7.9 (6.9–9.6)**		**0.007**	**7.1 (6.6–8.4)**		**8.0 (6.9–9.7)**		**0.014**
**Neutrophils/Lymphocytes (N/L) ratio (1–3)**	10.5 (7.1–14.5)		8.8 (5.5–14.6)		0.153	**10.4 (7.1–14.7)**		**8.7 (5.2–14.3)**		**0.05**
**Albumin**	3.3 (3.0–3.6)		3.3 (2.9–3.6)		0.557	3.3 (3.0–3.7)		3.3 (2.9–3.6)		0.451

LE, liver enzyme; IQR, interquartile range; ICU, intensive care unit; LDH, lactate dehydrogenase; CRP, C-reactive protein; HbA1c, glycated hemoglobin; N/L, Neutrophil/Lymphocyte ratio

The number of ICU admissions was 23 (25.5%) in the group that presented elevated liver enzymes and 36 (22.1%) in the group with normal LE during the initial period (*p* = 0.538). During the in-hospital follow-up, 32 (27.6%) patients in the group that presented elevated liver enzymes and 27 (19.7%) patients in the group with normal LE progressed to the ICU; this difference was not statistically significant (*p* = 0.179).

**Fig. 3:**
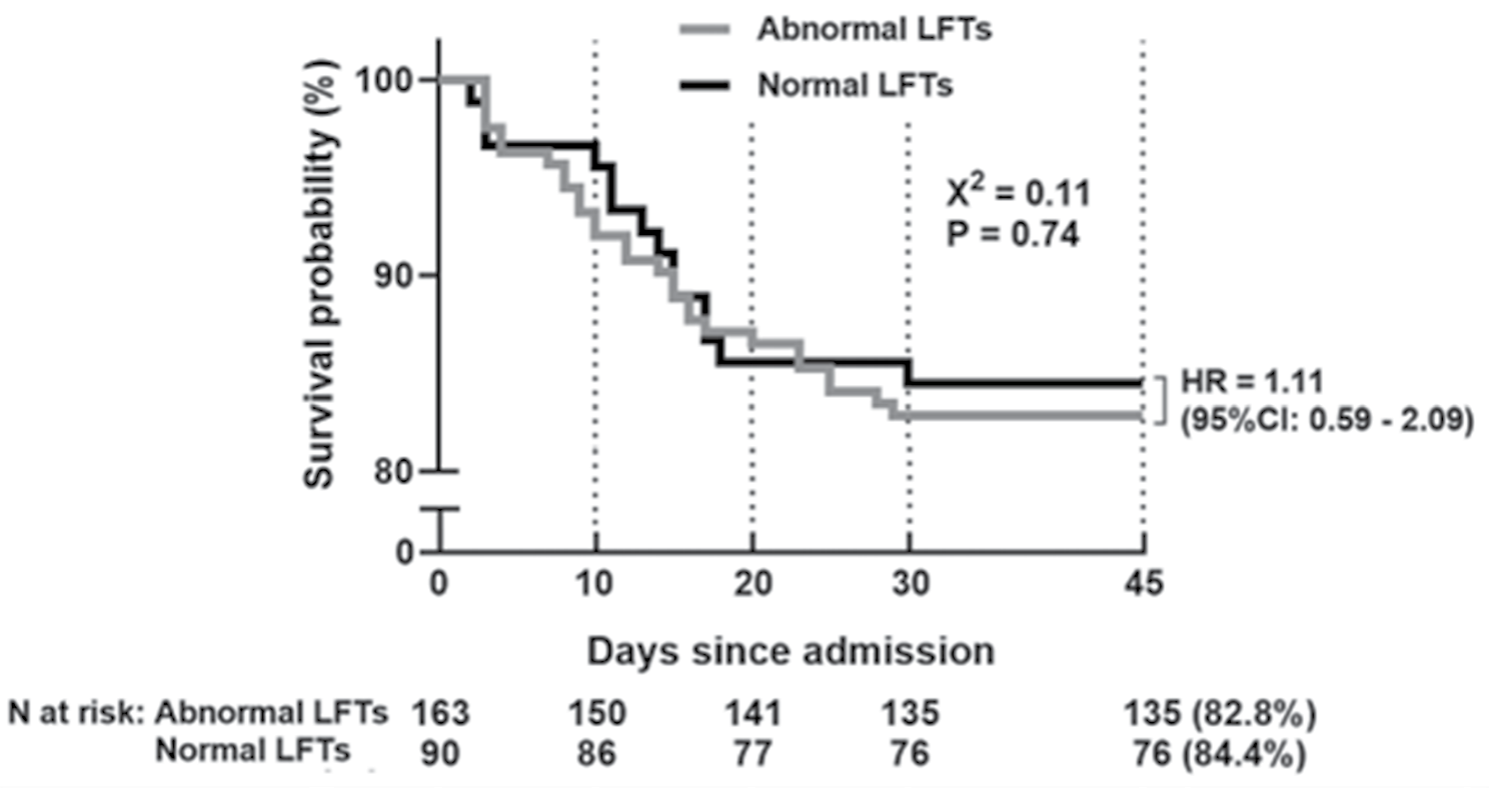
Kaplan‒Meier survival curve comparing patients with normal and abnormal liver function tests over 45 days. LFTs, liver function Tests; HR, hazard Ratio; CI, confidence interval

The analysis of mortality data revealed an incidence of 14 (15.5%) patients in the group that presented elevated liver enzymes versus 28 (17.1%) in the group with normal LE (*p* = 0.860). During the in-hospital follow-up, the death rate was 22 (18.9%) in the group with elevated LE and 20 (14.6%) in the group with normal LE; this difference was not statistically significant (*p* = 0.398). The 45-day survival probability did not significantly differ between patients with altered LE at admission (82.8%, *n* = 163) and those with normal values (84.4%, *n* = 90) (HR = 1.11; 95% CI: 0.59–2.09; *p* = 0.74) (Fig. 3).

## Discussion

The health crisis caused by the COVID-19 pandemic posed an unprecedented challenge to the global health system [11]. Early identification of the clinical or laboratory characteristics associated with the severity and mortality of COVID-19 is crucial for improved management and the development of clinical protocols [12]. This study described the incidence of gastrointestinal (GI) symptoms and liver enzyme alterations in 253 hospitalized COVID-19 patients in a tertiary hospital as well as their correlation with disease severity during a period when immunization against SARS-CoV-2 was not yet available.

In this prospective study, we observed that 19.37% (49 of 253) of hospitalized COVID-19 patients presented gastrointestinal (GI) symptoms, a rate that aligns with previous studies in Western populations reporting incidences of approximately 20% [13]. The prevalence of gastrointestinal manifestations in our cohort (19.37%) underscores the significant involvement of the digestive system in COVID-19 patients. In this context, maintaining high standards in gastroenterology and digestive endoscopy services is crucial for managing both acute and chronic gastrointestinal conditions during the pandemic. As discussed the development and status of digestive endoscopy have faced unique challenges in recent years, requiring continuous analysis of clinical census data to optimize patient care and diagnostic accuracy in tertiary centers [14]. In a meta-analysis that included 47 studies from international databases, Sultan et al. reported that the prevalence of gastrointestinal symptoms in COVID-19 patients ranged from 3% to 39.6%, with a lower incidence observed in studies conducted in Eastern countries [15]. The clinical management of hospitalized patients remains focused on the acute phase of COVID-19, particularly concerning the systemic inflammatory response and its multiorgan involvement [16].

Regarding imaging, chest computed tomography (CT) was the primary non-invasive modality used for patient evaluation at admission, despite the continuous development of new experimental lung imaging techniques [17]. Furthermore, in our cohort, gastrointestinal symptoms were not significantly associated with increased mortality, a finding that aligns with broader systematic reviews on the prognostic impact of digestive manifestations [18].

We did not find significant differences in mortality (16.2% in the GI symptoms group versus 18.3% in the non-GI symptoms group, *p* = 0.674) or ICU admission rates (20.4% in the GI symptoms group versus 24.2% in the non-GI symptoms group, *p* = 0.707) between patients with and without GI symptoms. This contrasts with some studies that suggested a correlation between GI symptoms and worse outcomes. For example, Jin et al. (2020), in an epidemiological study that evaluated 74 patients with gastrointestinal (GI) symptoms associated with COVID-19, reported a greater chance of developing severe disease and/or requiring critical care than did those without these symptoms (22.97% vs 8.14%, *p* < 0.001), even though there were no significant differences in radiological findings or inflammatory markers between the two groups, suggesting the possibility of an association between GI disorders and unfavorable outcomes in this patient group [8].

The diagnosis of COVID-19 cases presented significant challenges, particularly during the early phases of the pandemic when clinical presentations were highly variable. These difficulties are often exacerbated in clinical settings where patients present with fever of unknown origin, requiring a broad differential diagnosis to distinguish SARS-CoV-2 from other infectious or inflammatory etiologies. As highlighted understanding the distribution of causes for febrile illnesses is essential for accurate clinical management and resource allocation in tertiary hospitals facing public health crises [19].

In a systematic review and meta-analysis, Cheung et al. (2020) also reported a higher prevalence of GI symptoms in individuals who presented with more severe forms of the disease: 17.1% (95% CI 6.9–36.7) versus 11.8% (95% CI 4.1–29.1). However, there was still significant heterogeneity in the studies included in this meta-analysis (*p* < 0.001; I2 = 90.9% and I2 = 97.7%) [20]. Similarly, a meta-analysis that included 36 studies revealed that the presence of gastrointestinal symptoms was associated with an increase in disease severity [21, 22].

A study by Pan et al. revealed that patients with GI symptoms tend to have a more severe disease course, including a greater need for mechanical ventilation and ICU admission [23]. Our results, similar to those of Wan et al., indicated that although GI symptoms are common, they are not significantly correlated with COVID-19 mortality [24], indicating that in the studied population, GI symptoms do not significantly influence clinical outcomes in COVID-19 patients, which aligns with some studies that also reported the absence of a significant correlation between GI symptoms and disease severity.

Liver enzyme (LE) alterations were observed in 35.6% (90 of 253) of our cohort, which is consistent with other studies reporting hepatic enzyme abnormalities in COVID-19 patients. The prevalence of liver enzyme alterations in COVID-19 patients varies across studies. Zhang et al. reported that approximately 14% to 53% of patients presented elevations in liver enzymes, mainly ALT and AST [25]. Yu et al. reported a prevalence of ALT elevation ranging from 16% to 28%, whereas AST ranged from 20% to 37%, indicating that such findings are common in COVID-19 patients [26]. For example, Hajifathalian et al. (2020) reported biochemical evidence of liver injury in up to 62% of cases [27].

Zhang et al. and Cai et al. reported that there is a significant increase in liver enzymes in COVID-19 patients, especially those with severe disease. These studies suggest that elevated liver enzymes may be indicators of the severity of SARS-CoV-2 infection. Yu et al. also reported that elevations in ALT and AST are common and frequently correlated with disease severity, reinforcing the hypothesis that liver damage is associated with COVID-19 progression [20, 27, 28].

Abdulla et al. (2020) conducted a systematic review and meta-analysis of 25 studies to investigate COVID-19-induced liver injury and demonstrated that patients with COVID-19-induced liver injury had a worse prognosis, with higher ICU admission rates, mechanical ventilation needs, and mortality [29].

However, our study did not find a significant association between elevated LE and mortality rates (18.9% in the elevated LE group versus 14.6% in the normal LE group, *p* = 0.398) or ICU admission (27.6% in the elevated LE group versus 19.7% in the normal LE group, *p* = 0.179), diverging from findings of other researchers who identified elevated liver enzymes as independent predictors of severe disease and increased mortality. According to Lei et al., although liver enzyme elevation is observed, it is not directly correlated with mortality, indicating that other comorbidities and factors may influence these levels [30]. Wan et al. reported the absence of a significant correlation between elevated liver enzymes and disease severity, suggesting the need for further studies to clarify these relationships [31].

Although previous studies have explored gastrointestinal manifestations and liver enzyme abnormalities in patients with COVID-19, the present study provides additional and complementary information. Unlike earlier retrospective reports, our study prospectively evaluated hospitalized patients allowing standardized assessment at admission. Moreover, we examined the combined clinical and laboratory manifestations in relation to objective indicators of disease severity, providing integrated data on this specific population.

Singh et al. (2024), in a literature review that included 57 studies investigating the prevalence and mechanisms of liver injury in COVID-19 patients, reported that these elevations can occur due to various mechanisms, including direct injury by the virus and the effects of medications used in treatment, cautioning that they may not be reliable markers of infection severity [9].

Elevated liver enzymes measured at admission did not impact hospital stay duration and were similar in both groups: 6 (4–10) versus 6 (5–10) days (*p* = 0.830). However, the elevation of liver enzymes during hospitalization resulted in a longer hospital stay, with a median of 7 days (IQR: 5–12) compared with 5 days (IQR: 4–8.5) in patients who did not present this finding (*p* = 0.0016). This result is similar to that reported by Fan et al. [32]. However, Lv et al. questioned some conclusions of this study, attributing them to its retrospective design [33].

In a review of 43 retrospective studies conducted in 2020, Silva et al. evaluated aspects related to liver injury and concluded that the serum levels of liver enzymes are slightly elevated during SARS-CoV-2 infection. Most of the time, they are below twice the upper limit of normality; however, they observed that serum levels seem to increase with disease progression [9]. In 2021, another systematic review and meta-analysis of 14 studies combining data from 2871 patients reached a similar conclusion [34]. Both reviews reinforced the need for prospective studies to define the clinical relevance of liver injury in patients with COVID-19.

Our study showed that hemoglobin A1c (HbA1c) levels were lower in the group with abnormal liver enzymes (LE). During hospitalization, these levels were significantly lower than those in the group with normal LE. This differs from a cohort study conducted in the United States with 2006 patients, which reported that 20–30% of hospitalized COVID-19 patients had diabetes, and a significant portion of these patients also had elevated liver enzymes such as ALT and AST [35]. Although these results contradict those of previous studies in different populations, we can infer that in the studied group, prior metabolic factors related to glycemic metabolism do not seem to have contributed to the observed variations in liver enzymes.

Elevated liver enzyme (LE) and lactate dehydrogenase (LDH) levels in COVID-19 patients were significantly correlated. In the analyzed data, patients with abnormal LE had average LDH levels of 834 U/L, whereas those with normal LE had average LDH levels of 677 U/L at hospital admission (*p* < 0.0001). This statistically significant difference persisted throughout the hospitalization period, in agreement with previously published meta-analyses [36]. However, it was not possible to demonstrate a statistically significant association between mortality and unfavorable outcomes.

### Limitations

Although the total sample size (*n* = 253) exceeded the requirement for the primary cohort analysis (*n* = 158), the specific subgroup of patients presenting with gastrointestinal symptoms (*n* = 49) represents a smaller subset of the population. This reduction in the effective sample size for subgroup comparisons may have limited the statistical power to detect smaller, yet potentially clinical, differences in outcomes such as ICU admission or mortality between these groups. Consequently, while our findings accurately reflect this cohort, the absence of statistical significance in these specific associations should be interpreted within the context of the frequency of these manifestations in the study population

In the present study, liver involvement was assessed through serial measurements of aminotransferases during hospitalization, with the specific aim of characterizing acute liver enzyme alterations in patients with SARS-CoV-2 infection. Patients with known acute or chronic liver disease, chronic alcohol use, or exposure to hepatotoxic medications were excluded to minimize confounding by pre-existing hepatic conditions. Accordingly, the observed liver enzyme abnormalities are most consistent with acute systemic inflammation, viral-related injury, hypoxic stress, or treatment-associated effects rather than established chronic liver disease. Although we acknowledge that early or subclinical chronic liver disease cannot be entirely excluded in the absence of systematic pre-admission fibrosis assessment, this limitation is unlikely to have materially influenced our findings. Importantly, liver enzyme elevations in this cohort were not associated with ICU admission or mortality, and no progressive cholestatic pattern or signs of hepatic synthetic dysfunction were observed. These findings argue against a clinically relevant contribution of undiagnosed chronic liver disease to the outcomes assessed. Furthermore, previous studies have shown that early-stage liver disease may escape detection by routine liver biochemistry or conventional imaging [37], and that normal aminotransferase levels do not reliably exclude underlying liver pathology [38]. However, within the context of an acute-care, hospital-based study focused on short-term clinical outcomes, any potential misclassification would be non-differential and therefore unlikely to bias the observed associations.

## Conclusion

The presence of gastrointestinal symptoms (GI) was not correlated with disease severity or mortality in hospitalized patients with SARS-CoV-2. Patients who developed elevated liver enzymes during their hospital stay (LE) had a significantly longer length of stay. However, the elevation of LE was not significantly associated with more severe forms of the disease or mortality. This study contributes to the understanding of the relationships among gastrointestinal symptoms, elevated liver enzymes, and clinical outcomes in COVID-19 patients. Future studies with larger samples and prospective designs are needed to expand these findings and contribute to a better understanding of the various clinical characteristics of the disease.

## Data Availability

All data generated or analysed during this study are included in this published article.

## Abbreviations

ALT: Alanine transaminase
AST: Aspartate transaminase
CI: Confidence Interval
COVID-19: Coronavirus disease 2019
GI: Gastrointestinal
HR: Hazard Ratio
ICU: Intensive Care Unit
LE: Liver Enzymes
NEWS2: National Early Warning Score 2
rRT-qPCR: quantitative real-time reverse transcription polymerase chain reaction
SARS-CoV-2: Severe acute respiratory syndrome coronavirus 2
SOFA: Sepsis-Related Organ Failure Assessment
